# Effects of dental implant treatment on sleep quality in edentulous older people: A prospective cohort study

**DOI:** 10.4317/medoral.24214

**Published:** 2020-12-19

**Authors:** Fatih Karaaslan, Ongun Çelikkol, Ahu Dikilitaş, Umut Yiğit

**Affiliations:** 1 PhD DDS. Department of Periodontology, Faculty of Dentistry, Usak University, Turkey; 2 PhD DDS. Department of Prosthodontic Dentistry, Faculty of Dentistry, Usak University, Turkey

## Abstract

**Background:**

Edentulism and sleep disturbances are commonly seen among older people and cause serious negative effects on their daily lives. Edentulism can induce sleep problems by changing maxillo-mandibular anatomy and surrounding soft tissues. The effect of the treatment of complete edentulism on sleep disturbances is not sufficiently understood. The purpose of this cohort study is to detect how different treatment options affect sleep quality, daytime fatigue and sleep disorder breathing in totally edentulous elderly people.

**Material and Methods:**

Ninety-six individulas (50 male and 46 female) participated in this prospective cohort study. The patients were non-randomly assigned to three groups, fixed implant-supported prostheses (FP), removable implant-supported prostheses (RP) and conventional total prostheses (CP). The Pittsburgh Sleep Quality Index (PSQI), the Epworth Sleepiness Scale (ESS) and the STOP-Bang questionnaire were calculated before (T0) and one year after (T1) their prosthetic rehabilitations.

**Results:**

Although there was no statistically significant difference between groups in terms of mean PSQI (*p*=0.524), ESS (*p*=0.410) and STOP-Bang (*p*=0.697) scores at T0, there was a significant difference between groups in terms of mean PSQI (*p*=0.011), ESS (*p*=0.030) and STOP-Bang (*p*=0.024) scores at T1. The FP group, when compared to CP group was associated with significantly better scores in the PSQI (Δ = -3.399, 95% CI= -4.612 to -2.187), ESS (Δ = -1.663, 95% CI= -3.149 to -0.176) and STOP-Bang (Δ = -0.994, 95% CI= -1.592 to -0.397).

**Conclusions:**

Within the limitations of this study FP was associated with a positive influence on sleep disturbances. Randomized controlled trials will be needed to provide reliable inference on this association.

** Key words:**Dental implant, edentulism, older people, sleep disturbances.

## Introduction

The elderly population is rapidly growing, and life expectancy is increasing worldwide (1). Edentulism is one of the most common problems among older people, causing adverse effects on their health status and social well-being (2). Anatomical changes associated with edentulism, such as decreasing the vertical size of occlusion and alteration in the position of the hyoid bone or mandible induce sleep disturbances in older people (3,4).

Sleep disturbances are an important public health issue in the aging society; 50% of people over the age of 65 complain of poor sleep quality, excessive daytime fatigue and sleep disorder breathing (5,6). The gold standard for diagnosis of sleep disturbances is polysomnography (PSG) which is time consuming, impractical for clinical screening and requires sleep specialists not readily available at many hospitals (7,8). Therefore, several reliable and simple self-reporting questionnaires have been developed to quantify sleep disturbances. Three of the most widely used are the Pittsburgh Sleep Quality Index (PSQI), the Epworth Sleepiness Scale (ESS), and the STOP-Bang questionnaire. The PSQI was designed to measure sleep quality, the ESS was conceived to assess excessive daytime sleepiness and the STOP-Bang was developed to screen sleep disorder breathing (9-11).

It is recognized that edentulism may cause impaired chewing, swallowing and speaking, thus reducing quality of life (12). To resolve these problems, different types of prostheses have been developed, such as fixed implant-supported prostheses (FP), removable implant-supported prostheses (RP) and conventional total prostheses (CP), for the treatment of fully edentulous patients (13-15).

Several anatomical changes associated with edentulism can predispose a person to develop sleep complaints and increase the risk for sleep disturbances (16). Recent findings suggest that edentulism favours disturbed sleep and sleep disorder breathing (17-19). Although the relationship between edentulism and sleep disorders was reported, there have been no studies on how sleep disturbances change with treatment of edentulism. The aim of this study is to address that shortage of information by investigating the effects of FP, RP and CP in the treatment of completely edentulous elderly individuals using PSQI, ESS and STOP-Bang scores.

## Material and Methods

This study was conducted from October 2017 to June 2019 at the School of Dentistry of the University of Usak. Ninety-six individuals (50 male and 46 female) participated in the investigation. Individuals were informed about the purpose of the investigation and informed consent forms were signed. The study was conducted according to the Helsinki Declaration and ethical approval was procured from the Local Ethical Committee of Usak University.

Participants were included in the study according to the following inclusion criteria: 1) ≥ 65 years of age; 2) totally edentulous for at least four years; 3) do not consume alcohol or smoke tobacco; 4) capable of understanding the questionnaires used in the study; 5) no previous implant treatment. Exclusion criteria included: 1) uncontrolled diabetes mellitus; 2) any disease that may cause sleep disturbance (e.g., respiratory disease, airway infection, psychological or neurologic conditions; 3) use of insomnia medication; 4) implant failure during treatment; 5) body mass index (BMI) ≥ 30 kg/m2; 6) a change greater than 5% in BMI from the initial score.

Comprehensive clinical and radiographic examinations were performed by the same periodontist and prosthodontist in the patient assessment procedure. The patients were divided into three groups according to examination and patient decision. Each of patients were presented with three treatment options which were given below and the patients made a choice that can be influenced by patients’ financial status and education level (20).

1. Patients who requested dental implants to fixed implant-supported prostheses (FP group)

2. Patients with problems with mandibular prostheses and requested at least two mandibular implants for overdenture use. These patients had received mandibular implant-supported overdentures and conventional maxillary dentures. (RP group)

3. Patients who requested replacement of their complete removable dentures by conventional total prostheses. (CP group)

Patients were subjected to a standardized surgical protocol by the same periodontist (FK) using a single implant system. After the osseointegration period, the prosthetic rehabilitation was completed by the same prosthodontist (OC). Other investigators administered the questionnaires.

The questionnaires were based on demographic information (age, gender, education level and monthly income), PSQI, ESS and STOP-BANG formats. Patients’ education level was differentiated as primary school, secondary school, high school, or university. Monthly income of participants was categorized as low (<5.000TL), middle (5.000-10.000 TL) and high (>10.000TL).

Before undertaking prosthetic rehabilitation (T0) and one year after receiving prostheses (T1), all patients were assessed by questionnaire. All questionnaires were completed in face-to-face interviews. The questionnaires were administered to each patient at T0 by an investigator (UY) who was blinded to the patient’s group. The same questionnaires were administered to each patient at T1 by another investigator (AD) who was also blinded to the patient’s group. The questionnaires were filled out in the same environment and conditions for all patients at both interviews.

The PSQI is a reliable self-reporting questionnaire that includes seven clinical domain scores, each ranging from 0 to 3. The total domain score gives the mean global PSQI score that varies between 0–21. A mean PSQI global score ≥ 5 represents poor sleep quality and sleep disturbances (21).

The ESS includes eight questionnaires that evaluate subjective daytime sleepiness (10). Each questionnaire ranges on a scale of 0–3 that collects information about how often and in what situations an individual falls asleep. The mean global ESS score ranges from 0 to 24; any score ≥ 10 suggests a high risk of falling sleep (7).

STOP-Bang questionnaires consider snoring, tiredness, observed apnoea, high blood pressure, high BMI, age, neck circumference and gender, and were developed as a screening tool for sleep disorder breathing in preoperative clinics. The questionnaire includes eight yes/no questions. Yes to 3 or more questions is considered a high risk for sleep disorder breathing (11).

Data analysis was performed using Statistical Package software, version 17.0 (SPSS Inc., Chicago, IL, USA). Kolmogorov-Smirnov and Shapiro Wilks tests were used to check the normality of data. The gender distribution of the groups was compared by using Chi square test and the mean age of the groups was compared by using One-Way Anova test. The education level and monthly income of the groups was compared by using Chi square test. The weight of the groups was compared by using One-Way Anova test. The adjusted means and mean differences were analyzed by using Univariate analysis. The mean scores of PSQI, ESS and STOP-Bang at T0 and T1 for groups were analyzed by using Ancova test with adjustment for age, gender, education level, monthly income and weight. Bonferroni test was performed when there was a difference. The statistical significance level was set at 0.05.

## Results

The study group was comprised of 50 male (52.08 %) and 46 female (47.92 %). There was no statistically significant difference between groups in terms of gender distruption (*p*>0.05, Chi square test) (Table 1).

**Table 1:**
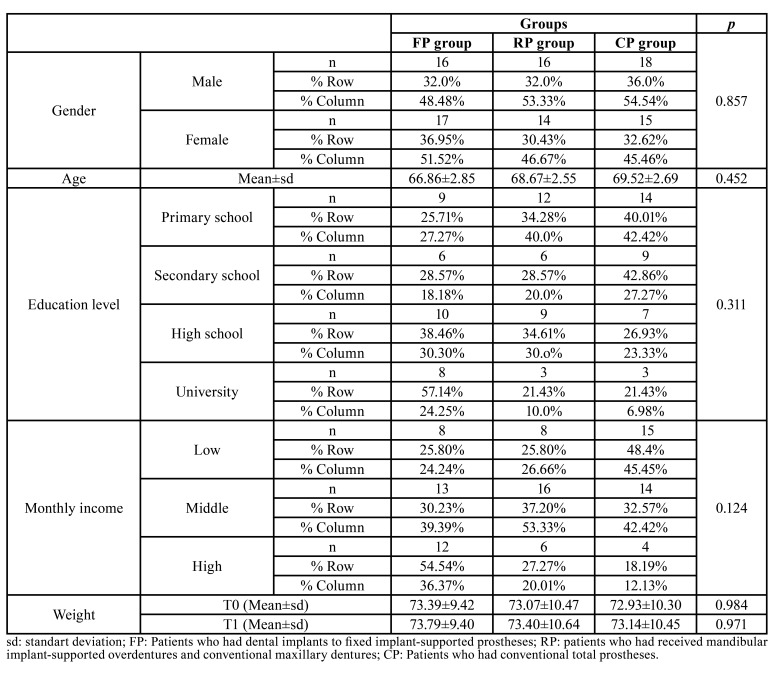
The baseline socio-demographic characteristics of individuals.

The age of the individuals ranged from 65 to 75 years, with a mean of 69.01 ± 2.69 years. There was no statistically significant difference between groups in terms of mean age (*p*>0.05, One-way Anova test) (Table 1).

Although, the education level and monthly income of individulas in FP group were higher than individuals in the RP and CP groups, this difference was not significant (*p*>0.05, Chi square test) (Table 1). There was no significant difference between the groups in terms of individuals' T0 and T1 mean weight (*p*>0.05, One-way Anova test) (Table 1).

Although there was no statistically significant difference between groups in terms of mean PSQI, ESS and STOP-Bang scores at T0 (*p*>0.05, Ancova test), there was a significant difference between groups in terms of mean PSQI, ESS and STOP-Bang scores at T1 (*p*<0.05, Ancova test) (Table 2).

The Bonferroni test also indicated the significant difference between groups in terms of mean PSQI, ESS and STOP-Bang scores at T1 (Table 3). Furthermore, the mean differences of PSQI, ESS and STOP-Bang scores between groups at T0 and T1 were shown in Table 4.

**Table 2:**
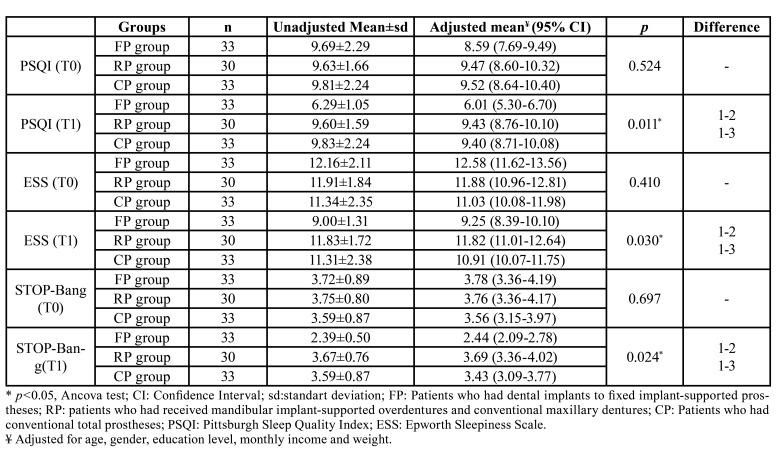
The difference between groups in terms of mean T0 and T1 scores.

**Table 3:**
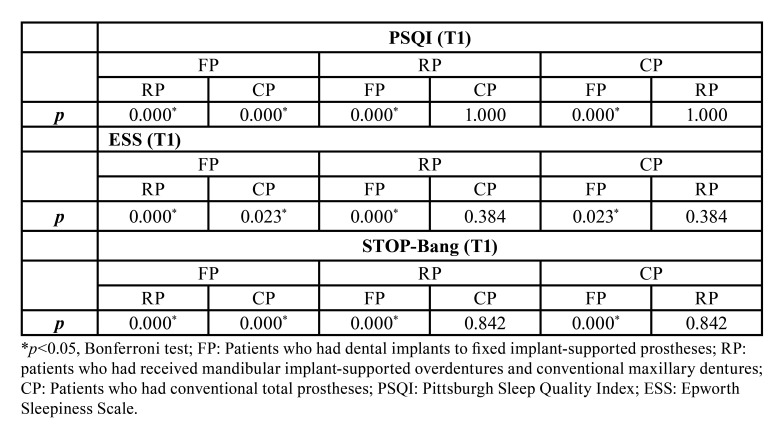
Bonferroni test at T1 scores of PSQI, ESS and STOP-Bang.

**Table 4:**
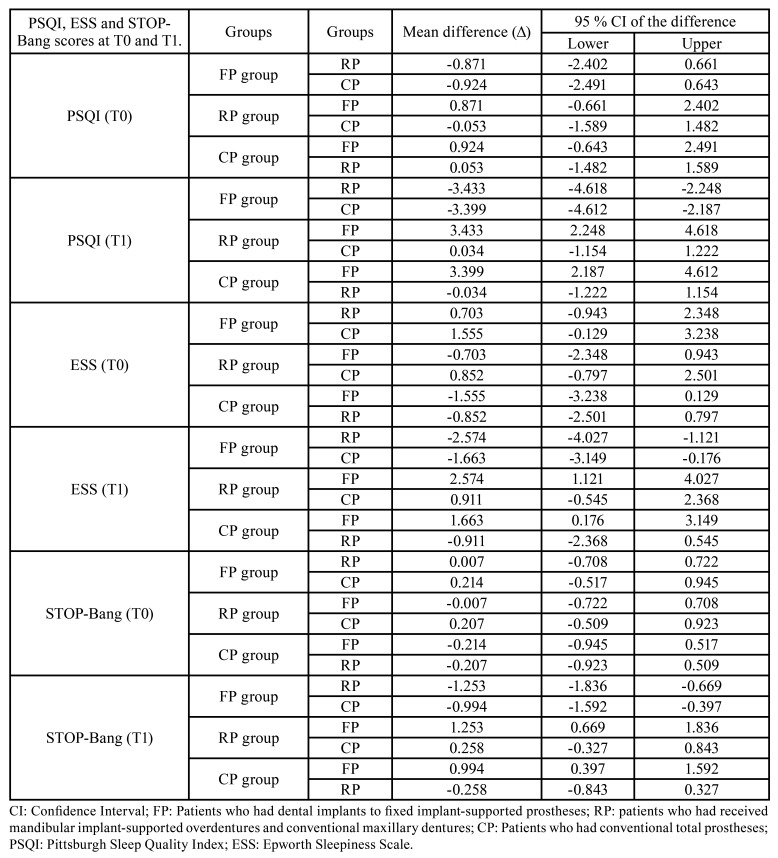
The mean difference of PSQI, ESS and STOP-Bang scores between groups at T0 and T1.

## Discussion

The present study demonstrates that FP was associated with a beneficial effect on sleep disturbances of edentulous elderly people. These study findings cannot be directly compared with any earlier similar investigations because, to the best of our knowledge, this is the first investigation conducted on how FP affects sleep disturbances. In particular, we highlight these possibilities: FP leads to an increase in oral innervation, a gain in oropharyngeal coordination and enhanced osseoperception by treating edentulism-induced neuromuscular dysfunction (22). These mechanisms may reduce sleep disturbances caused by edentulism. Another explanation is that masticatory muscle activity increases during sleeping (22). More jaw elevator muscle activity was reported among edentulous people. FP may increase the pharyngeal patency by interrupting the activation of the elevating muscles which may pull the mandible up and forward. This positive change associated with FP results in a beneficial effect on the upper airway decreasing collapses (17). It can also be hypothesized that FP has an effect similar to occlusal splints in muscle relaxing and contributes to an improvement in upper airway collapsibility by moving the mandible down and back (17). Moreover, the posterior airway space and retropharyngeal space may increase after reconstructing the vertical dimension of occlusion with FP (23). Mandibular edentulism may lead to tongue retraction and hypertrophy causing retroglossal space obstruction (24,25). It can also be speculated that FP rehabilitates normal tongue posture causing a widening of the upper airway and contributing to a reduction in sleep disorder breathing (25).

In the RP and CP groups, these edentulism treatment options were not associated with a beneficial effect on mean scores of PSQI, ESS and STOP-Bang. This lack of an association could be due to the fact that RP and CP reconstruct a normal maxilla-mandibular relationship rather than guiding mandibular protrusion to prevent upper airway collapse associated with the anatomical changes caused by edentulism (26). We also speculate that complete dentures are not as effective as fixed prostheses in the restoration of the vertical dimension of occlusion.

In this study, removal of RP and CP during the night was recommended to avoid inflammation of oral mucosa and associated irritations. In this regard, some studies have suggested that wearing complete dentures during sleep has a beneficial effect on sleep complaints (18,27), whereas other studies have found no significant difference between wearing and not wearing complete dentures (28,29). The lack of association in the RP and CP groups with beneficial effect on mean scores of PSQI, ESS and STOP-Bang might be due to the fact that patients were not wearing removable protheses during sleep in this study. Therefore, PSQI, ESS and STOP-Bang scores at T0 and T1 were similar in RP and CP groups. Although the effect of nocturnal wearing of prostheses on sleep disturbances is still not fully understood and should be further investigated, it should be kept in mind that long-term nocturnal wearing of complete dentures causes chronic mucosal inflammation by reducing the protective effect of saliva and oxygenation on the oral mucosa. Due to this factor, nocturnal wearing of dentures did not pass as more than a temporary solution. In this context, continuous use of FP may be associated with more effective treatment of sleep disturbances, especially if it is not necessary to remove it at night for sleep.

In addition, we investigated the effect of socioeconomic status on treatment choice. Treatment decisions can be performed depending on not only clinical examination and dentist’s opinion but also patient’s decision which can be influenced by patients education level and monthly income (30). In this study, although there is no significant difference between groups in terms of education level and monthly income, individuals who prefer FP treatment option have higher education level and monthly income. Unlike other studies, the reason for the lack of difference between education level and monthly income with treatment options may be fear of surgery, having the same socio-cultural structure and fear of unknown side effects of implant treatment (30).

The mean baseline scores of PSQI, ESS and STOP-Bang questionnaires may differ from other studies’ baseline scores. This can be explained as mean scores might vary according to different cultures. Using questionnaires to identify sleep disturbances is a limitation in this study, although these questionnaires are known to be highly sensitive, reliable and effective screening tools for sleep disturbances.

## Conclusions

The results of this study indicate that treatment of edentulism with FP was associated with a positive effect on sleep disturbances of older people, when compared to RP and CP treatment. Randomized controlled trials are needed to determine the causality of this association between treatment of edentulism and sleep complaints of older people.
